# Manifestation of Adult children of patients having Alzheimer’s dementia

**DOI:** 10.1002/alz.084431

**Published:** 2025-01-09

**Authors:** Kuan‐Ying Li, Yuan‐Han Yang

**Affiliations:** ^1^ Kaohsiung Medical University, Kaohsiung Taiwan; ^2^ Kaohsiung Municipal Ta‐Tung Hospital, Kaohsiung Taiwan

## Abstract

**Background:**

Individuals with his parents having Alzheimer’s dementia (AD) have a 4 to 10 times higher risk of developing AD compared to those without family history. The increasing risks of developing AD may result from several underlying mechanisms to be clarified for further strategies of management. We are going to report the demographic and biological characteristics of adult children (AC) of AD parents cohort with its clinical significances in Taiwan.

**Method:**

AC of AD patients age from 50‐79 years old were recruited from the neurological outpatient department of Kaohsiung Municipal Ta‐Tung Hospital, Taiwan. The paired individuals, adult children with his/her parents were received annual following up in clinical, neuropsychological, and neuroimaging including PET/CT with 18F‐Florbetaben (FBB) imaging session, using DiscoveryTM MI DR PET/CT system (General Electric Medical Systems, Waukesha, Wisconsin, USA) examinations and assays of blood for genetic and protein analyses. This study was approved by Kaohsiung Medical University Hospital Institutional Review Board. Written informed consent was obtained from all participants before including in this study.

**Result:**

63 AC with age 59.3 ± 5.8 years, female predominant (76.2%), baseline MMSE 28.3 ± 1.8, CASI 94.7 ± 3.4, and having at least one of APOE e4 allele 34.9%, have recruited. 19 voluntary participants (25.4%) completed 18F‐FBB PET. 9 of 19 (47.4%) were classified as amyloid‐positive based on visual assessment, rated by a senior board‐certificated nuclear medicine physician with experience in interpretation of 18F‐FBB PET who evaluated all images according to the guideline of 18F‐FBB PET interpretation, blind to all clinical information.

**Conclusion:**

AC of AD patients have higher proportion of having APOE4 allele and higher proportion of having amyloid PET (+). It may imply AC does increase the risk of developing AD, and AC with amyloid PET (+) could be preclinical AD.

